# Dual inversion recovery late gadolinium enhancement for more accurate infarct size determination: a histological validation study

**DOI:** 10.1186/1532-429X-15-S1-E50

**Published:** 2013-01-30

**Authors:** Anne Yoon Krogh Grøndal, Sarah A Peel, Lars Bloch, Esben S Hansen, Steen Fjord, Jesper L Hønge, Rene M Botnar, Won Yong Kim, William P Paaske

**Affiliations:** 1 Department of Cardiothoracic and Vascular Surgery, Aarhus University Hospital Skejby, Aarhus, Denmark; 2 The MR Research Centre, Aarhus University Hospital Skejby, Aarhus, Denmark; 3 Department of Cardiology, Aarhus University Hospital Skejby, Aarhus, Denmark; 4 Imaging Sciences and Bioengineering King's College London, King's College London, London, UK

## Background

Cardiovascular magnetic resonance (CMR) deploying late gadolinium-enhanced inversion-recovery (IR) sequence is today's standard reference for myocardial infarction evaluation. However, the conventional IR sequence can yield poor image contrast between infarct and intracavity blood pool which complicates precise endocardial border delineation. This compromises accurate infarct size determination and small subendocardial infarct detection. Peel* et al.* 2012 found that a dual IR prepulse outperformed conventional IR in infarct visualization, scar-to-blood contrast and expert consistency, but this novel technique has not been histopathologically validated. This study sought to compare dual IR prepulse and conventional IR sequences with histopathological findings in an animal model of reperfused acute myocardial infarction.

## Methods

Under general anaesthesia, ischaemia-reperfusion injury was induced in nine pigs (40 kg) by 40-minute balloon occlusion in LAD followed by reperfusion. One day post-injury, CMR was performed using conventional IR and dual IR sequences at 1.5T. After this, the pigs were euthanized. Their hearts were then explanted, axially cut and incubated in a 2,3,5-triphenyltetrazolium chloride (TTC) solution (10%). CMR images were matched to the corresponding histopathology and the infarct sizes were compared.

## Results

**Figure 1:**
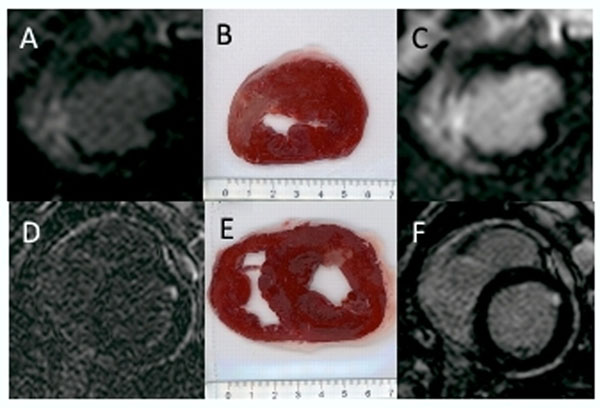
Short-axis CMR and corresponding pathology obtained one day following ischaemia-reperfusion injury in two different pigs. The upper panel showing dual IR (A), corresponding pathology (B) and conventional IR (C) from the same pig. The lower panel showing dual IR (D), corresponding pathology (E) and conventional IR (F) from the same pig.

**Figure 2:**
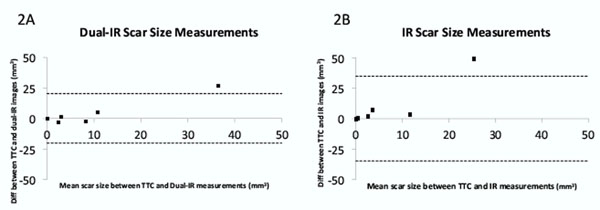
Bland-Altman plot comparing infarct size measurements determined by dual IR and TTC (A) and by IR and TTC (B). Solid line indicates the mean of the differences, and dashed lines indicate 1.96 standard deviations above and below the mean of the differences.

Nine pigs underwent the ischaemia-reperfusion procedure. Two were excluded due to ECG-triggering problems. Five out of the remaining seven pigs showed myocardial infarction after TTC staining. Both dual IR and conventional IR confirmed infarctions in all five pigs. Short-axis dual IR images show improved blood suppression compared with IR images and show good correlation with TTC images (Figure 1). In Bland-Altman analysis, scar size measurements made on dual IR images had better correlation with histology compared with the IR images (Figure 2).

## Conclusions

In this preliminary, experimental study, the dual IR prepulse resulted in more accurate myocardial infarct size determination compared with conventional IR sequence owing to better blood suppression.

## Funding

King's College London.

